# Predicting pregnancy test results after embryo transfer by image feature extraction and analysis using machine learning

**DOI:** 10.1038/s41598-020-61357-9

**Published:** 2020-03-10

**Authors:** Alejandro Chavez-Badiola, Adolfo Flores-Saiffe Farias, Gerardo Mendizabal-Ruiz, Rodolfo Garcia-Sanchez, Andrew J. Drakeley, Juan Paulo Garcia-Sandoval

**Affiliations:** 1 New Hope Fertility Center Mexico, Research and Development, Guadalajara, PC 44630 Mexico; 2 Universidad de Guadalajara, Departamento de Ciencias Computacionales, Guadalajara, PC 44430 Mexico; 3 Hewitt Centre for Reproductive Medicine, Clinical Director, Liverpool, PC L8 7SS United Kingdom; 4 Universidad de Guadalajara, Departamento de Ingeniería Química, Guadalajara, PC 44430 Mexico

**Keywords:** Biotechnology, Pregnancy outcome, Preclinical research

## Abstract

Assessing the viability of a blastosyst is still empirical and non-reproducible nowadays. We developed an algorithm based on artificial vision and machine learning (and other classifiers) that predicts pregnancy using the beta human chorionic gonadotropin (b-hCG) test from both the morphology of an embryo and the age of the patients. We employed two high-quality databases with known pregnancy outcomes (*n* = 221). We created a system consisting of different classifiers that is feed with novel morphometric features extracted from the digital micrographs, along with other non-morphometric data to predict pregnancy. It was evaluated using five different classifiers: probabilistic bayesian, Support Vector Machines (SVM), deep neural network, decision tree, and Random Forest (RF), using a k-fold cross validation to assess the model’s generalization capabilities. In the database A, the SVM classifier achieved an F1 score of 0.74, and AUC of 0.77. In the database B the RF classifier obtained a F1 score of 0.71, and AUC of 0.75. Our results suggest that the system is able to predict a positive pregnancy test from a single digital image, offering a novel approach with the advantages of using a small database, being highly adaptable to different laboratory settings, and easy integration into clinical practice.

## Introduction

Since the inception of IVF, morphology has been the gold standard for embryo selection. Despite the existence of clear and detailed guidelines for morphology-based classifications, blastocyst morphology assessment remains an artisanal technique, which lacks objectivity and reproducibility, limiting its overall predictive value. Furthermore, current scoring systems for human blastocysts focus on only three parameters (*i.e*., degree of expansion, quality of inner cell mass, and quality of trophectoderm), while other features that may have an impact on implantation potential are mostly disregarded^1,2^.

The challenges inherent in image-based diagnosis and decision-making are not unique to reproductive medicine. Efforts directed at improving accuracy and standardization of image analysis through development of computer-aided tools have recently gained attention in other medical fields^3–5^, including dermatology and oncology, where deep learning techniques and architectures are in current use for image analysis and to assist diagnosis^6,7^.

The field of reproductive medicine has also ventured into the world of artificial intelligence (AI)^8^ but, as yet with somewhat limited clinical impact. The quantitative evaluation of embryo characteristics with digital imaging systems has become an active research area^9,10^, and efforts have been made to improve objectivity during the embryo selection process by combining multiple morphological parameters and morphokinetic analysis in mathematical models, aiming to generate predictive capabilities for development potential and implantation. Such models, however, are often limited to initial development stages of the embryo, dependent on currently existing classifications, or require sophisticated time-lapse microscopy systems^11–14^.

In this study, we present a computer-aided classification system designed to identify new and objective morphological parameters at the blastocyst stage of *in-vitro* development. By using an independent set of transferred embryos with known outcomes, we validated the ability of this system to predict the outcome defined as beta-human chorionic gonadotropin (b-hCG) results.

## Results

A total of 221 transferred blastocysts were included in this study: 134 from dbA; and 87 from dbB. Overall positive pregnancy rate per embryo transfer was 54.1% (95% CI 47.3–60.8%). The mean age at oocyte retrieval was 34.4 years. Table 1 shows the differences and similarities between the two data-sets.

**Table 1:** Databases description. ^*^4 unknown.

Feature	dbA	dbB
Objectives (20X, 40X)	(90, 44)	4, 83)
Donated oocytes (%)	16.92%^*^	12.64%
Mean oocyte age (STD)	34.11 (6.09)	34.93 (4.96)
Embryos expanding	62.69%	78.17%
Embryos hatching	32.83%	17.23%
Embryos hatched	4.48%	4.60%
b-hCG $\ge 20\ mUI$/$mL$ (%)	52.98%	56.32%
Proportion of fresh oocytes	13.43%	6.89%

**Figure 1:**
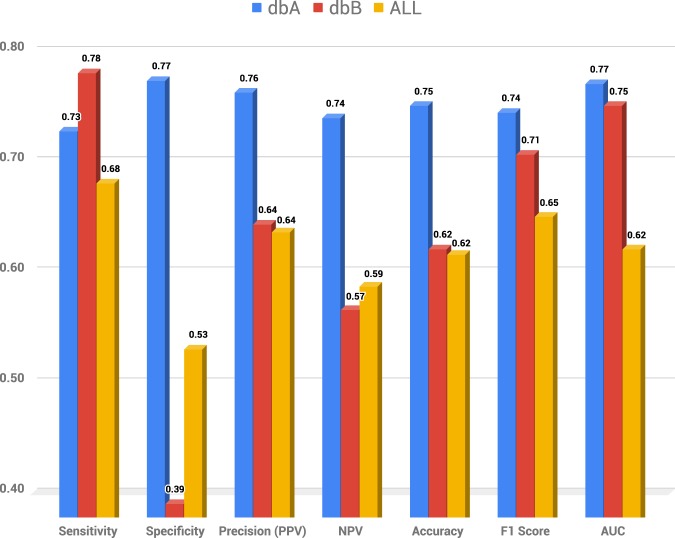
Comparative chart of the best model for each database. The dbA and ALL databases obtained the best F1 score using the Support Vector Machine model, and the dbB database using the Random Forest model. AUC - Area Under the receiver operating characteristic Curve.

Based on the F1 score, the SVM was the model that obtained the best performance in dbA and ALL databases, while Random Forest performed better in the dbB data. Figure 1 shows sensitivity, specificity, precision, accuracy, F1 score, and AUC for the best model for the three data-sets. False-positive rate (FPR) and False-negative rate (FNR) were also computed for all three data-sets (See Table 2).

**Table 2:** False positive and negative rates.

	dbA	dbB	ALL
FPR	0.23	0.61	0.47
FNR	0.27	0.22	0.32

To ensure our results are not a consequence of over-fitting, we randomized the pregnancy test results overall samples and tested the models. For the dbA database, the SVM obtained an F1 of 0.52; the dbB database obtained an F1 of 0.52 on the RF model, and the ALL database obtained an F1 of 0.57 on the SVM model.

## Discussion

The results in this study suggest software prototype AIR E, can successfully extract features from individual blastocyst micrographs to feed its embedded algorithm and then predict b-hCG test results.

For sensitivity, the dbB performed slightly better than dbB (See Fig. 1). However, in specificity, precision, and accuracy, dbA overcame dbB, because of the high FPR of the later (See Fig. 1 and Table 2). The main differences between these two data-sets are the objective used in the microscopy and the proportion of expanding/hatching blastocysts, which suggests that these variables could be influencing the specificity. Still, we have not excluded the possibility of other confounding factors that might be affecting the outcome.

Using all the data-set, we obtained a sensitivity of 0.68 when the algorithm labels a blastocyst will result in a positive b-hCG. This result is close to the success rate expected from transferring euploid blastocysts following a pre-implantation genetic test for aneuploidies (PGT-A)^15^. However, we emphasize that the present work is only a pilot study.

Image-based diagnoses and subsequent decision- making processes are challenging in that unless based on objective and standardized criteria, they are likely to jeopardize prognostic accuracy and efficiency of decision making. Current blastocyst selection is in just such a predicament since most accepted classifications are based on subjective assessments during an artisanal process performed by embryologists. Furthermore, and perhaps in the interest of simplicity, current non- morphokinetic classifications are based on characteristics that require no measurements. They are therefore subjective, disregarding variables or characteristics that cannot be consistently identified by the naked eye, but which could potentially be reflective of development potential.

By contrast, computer-aided image processing tools can identify, in an automated, objective, and replicable fashion, key image characteristics beyond what is recognizable by the “naked eye”. Thus, these technologies could help transform current blastocyst classification from subjective and morphology-based, to an automated, objective, and standardized image processing software. In reproductive medicine, efforts have been directed towards implementing computational tools and artificial intelligence software for the blastocyst selection process, but until recently most of these efforts have either focused on predicting blastocyst formation^8^ or have been designed to fit current embryo classifications^10,16,17^, moving a step further into automation, but failing to link embryo classification to viability prediction.

Our current software prototype, on the other hand, takes a different approach by disregarding existing classifications and linking independent and new variables to prognosis. By bypassing “standard” morphology, the prognosis determined by the algorithm could allow an embryo ranking system and a more straightforward decision-making process during embryo selection for transfer.

In the same line as our study, and to associate morphological characteristics extracted from micrographs to prognosis, Tran *et al*. (2019) trained an algorithm using 8836 embryos to predict fetal heart pregnancy using a time-lapse incubator^14^. However and despite the high AUC obtained (0.93), these results should be taken with caution, since they used a highly unbalanced data-set (92% of negative) that could be deceptive of the model performance, especially when the AUC is the only reported measure and this test has been described as sub-optimal for such data-sets^18,19^. Besides, the authors included embryos with “failed or abnormal fertilization and grossly abnormal morphology” into the negative group, which could also mislead the reported measure because they did not reach the blastocyst stage and therefore the outcome was known before the embryo transfer even without the need of an algorithm.

Another cornerstone to Tran’s, Rocha’s, and Khosravi’s approach is that input information came from micrographs obtained from the EmbryoScope^10,14,17^, allowing the investigators to standardize variables related to the use of different imaging protocols and settings (*e.g*., camera type, objectives, microscope type, etc.). This advantage, however, seems to be tempered by a lack of flexibility, for example, the inability to classify hatching embryos, and even more critical, a limitation in the form of a requirement for expensive time-lapse equipment.

Once again, we decided against a tempting standardized approach but instead trained our algorithm for image extraction and processing after identifying and automatically standardizing for imaging variables. In this way, we aimed to generate a computational tool more natural to implement in clinical practice, with equipment currently available in most IVF laboratories.

Approaches to predict blastocyst quality, like the ones discussed above, rely on the need for large sample size due to their bottom-up approach. AIR E, on the other hand, achieves high accuracy using a relatively small sample size due to the software’s ability first to extract features from the images and then to feed these to the classifier (top-down approach). This top-down approach allows AIR E to learn from each microscope and thus to make it highly flexible and adaptable to multiple clinics and conditions.

To the best of our knowledge, this is the first AI classifier trained and evaluated on static micrographs of blastocyst-stage embryos of single transferred embryos that predicts b-hCG.

We acknowledge classification algorithms thrive on numbers, and therefore, the authors are currently pursuing a study with a larger sample size with the final goal of developing a blastocyst ranking system based on prognosis. We believe the implementation of this program could impact on standardization of embryologists decisions, assist towards IVF laboratory automation and improved efficiency, assist with research protocols where embryo grading is the core of the study, and more important, to reduce time to pregnancy and live birth by supporting the selection of the best embryo available for transfer each cycle. Still, we believe a study design like the one we present, is not strong enough to attempt reaching such conclusions and a prospective study, as the subject of future work, would validate the clinical application of the proposed model.

## Methods

We performed a morphometric analysis of micrographs of blastocysts transferred as single embryos between 2015 and 2019 at two fertility centers in Mexico (dbA and dbB). The databases were tested both independently and combined (data-set named ALL). A total of 221 blastocysts were photographed on day 5–6 after insemination using either of two inverted microscopes: Olympus IX71 (dbA); or Olympus IX73 (dbB), both with Hoffmann modulation contrast, a digital camera and a Hamilton Thorne ZILOS-tkï¿½ő Laser camera. The complete data-set consisted of images taken at 20x (total magnification 200x), and 40x (total magnification 400x). Embryologists did not adjust light features and calibration in a standard fashion. The later means that the observed features are developed from varying picture settings akin to a large variation similar to using different equipment. Each blastocyst was photographed one time only, with a focus on the zona pellucida and trophectoderm.

  Table 2 describes relevant metadata of the databases, including the objectives used, the oocyte source, the mean age of the oocyte, the embryo stage, the proportion of fresh samples, and b-hCG $\ge$ 20.

All transfers were single embryo transfers of blastocysts (180 in diameter as measured before transfer), performed following previously published protocols^20^. In brief, fresh transfers were performed five days following oocyte retrieval with luteal support given as 400 mg vaginal progesterone (Geslutin, Asofarma, USA), every 12 hours until the pregnancy test. For frozen embryo transfer, endometrial preparation was started with 4 mg of oral estradiol (Primogyn, Schering AG, Colombia) from day 3 of the menstrual period and supplemented with 400 mg vaginal progesterone (Geslutin, Asofarma, USA) every 12 hours starting on day 14–16 of the cycle; both continued until the pregnancy test. Blastocysts were thawed 2–4 hours before embryo transfer on day +6 from the beginning of luteal phase support. Seven days following embryo transfer, a quantitative b-hCG assay was performed. Beta hCG levels $\ge 20\ mUI$/$mL$ were interpreted as positive.

### Image analysis and statistical assessment

**Figure 2:**
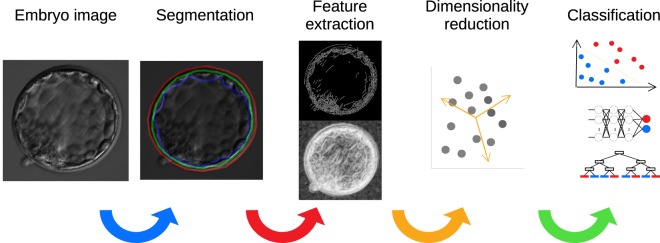
Overview of the AIR E pipeline. Once the embryo image is taken, it is passed through Segmentation, Feature Extraction, Feature Selection, and Classification.

An image processing algorithm available in the AIR E prototype system was used to analyze all 221 blastocysts included in the study (see Fig. 2). Using AIR E, two embryologists manually marked the boundaries (delimited) of the zona pellucida and the trophectoderm, as it is observed in the Fig. 2 (Segmentation), allowing the algorithm to extract and compute a total of 24 features from each image by performing pattern variation analysis of pixel intensities, and other relevant morphological properties (*e.g*., areas and perimeters). To homogenize images, AIR E transformed all the features from pixels to micrometers according to the calibration data of each microscope. In a step-ward fashion, AIR E extracts the following features: perimeter, area, pixel information (obtained by applying an entropy filter to the embryo image), the standard deviation of the pixel information, and the amount of boarders found within the image (obtained through the canny algorithm). These metrics were obtained independently for the group of pixels labeled as zona pellucida, trophectoderm and inner area (blastocoel and inner cell mass). The feature extraction step is followed by principal components analysis (PCA) on all of the features as a pre-processing step, obtaining 14 components which represent 99% of the variability in the data-set. Patients’ age was included in AIR E as the only feature not based on image analysis.

AIR E performed training of five supervised ML models to classify samples with b-hCG $\ge$ 20 $mUI$/$mL$. The selected ML models are representative of the different types of classifiers: (1) Naive Bayes, (2) v - Support Vector Machine (v-SVR model for minimization of the errors, and RBF kernel with $g=0.14$), (3) deep Neural Network (three layers of 30 units each and one with 10 units, ReLu activation function, Adam solver, and L2-Regularization of 0.0001), (4) Decision Tree (unrestricted), and (5) Random Forest (100 trees). To assess the generalization capability of the classification models and avoid over-fitting, we used stratified 10-fold cross-validation as suggested in previous studies^21^, and the reported measures are the average of the results of each fold. To assess the results, we computed the sensitivity, specificity, precision, accuracy, F1, and Area Under the receiver operating characteristic curve (AUC).

### Ethical considerations

All patients signed appropriate consents for treatment according to internal protocols and the use of the images of the blastocysts for research purposes. The data has been anonymised upon collection. None of the authors accessed the patients information during the data analysis. The data has restricted access. Since this study was a retrospective study for image processing validation with no additional intervention, IRB (CONBIOETICA 09-CEI-00120170131) approval was waived (RA 2018-01) by the New Hope Fertility Center Mexico’s Ethics Committee. All methods were performed in accordance with the relevant guidelines and regulations.

All the data employed for this study was anonymized, and the authors did not have access to any information that could reveal the patient’s identities during the study.

The data that support the findings of this study are available on request from the corresponding author ACB. The data are not publicly available due to them containing information that could compromise participant consent. The authors did not have access to any information that could reveal the patient’s identities during the study.
